# Even low alcohol concentrations affect obstacle avoidance reactions in healthy senior individuals

**DOI:** 10.1186/1756-0500-3-243

**Published:** 2010-09-23

**Authors:** Judith Hegeman, Vivian Weerdesteyn, Bart JF van den Bemt, Bart Nienhuis, Jacques van Limbeek, Jacques Duysens

**Affiliations:** 1 Department of Research, Development & Education, Sint Maartenskliniek, Nijmegen, The Netherlands; 2 Institute for Fundamental and Clinical Human Movement Sciences (IFKB), Amsterdam, The Netherlands; 3 Department of Rehabilitation, Radboud University Nijmegen Medical Centre, Nijmegen, The Netherlands; 4 Department of Pharmacy, Sint Maartenskliniek, Nijmegen, The Netherlands; 5 Faculty of Kinesiology and Rehabilitation Sciences, Department of Biomedical Kinesiology, Leuven, Belgium

## Abstract

**Background:**

Alcohol is a commonly used social drug and driving under influence is a well-established risk factor for traffic accidents[1]. To improve road safety, legal limits are set for blood alcohol concentration (BAC) and driving, usually at 0.05% (most European countries) or 0.08% (most US states, Canada and UK). In contrast, for walking there are no legal limits, yet there are numerous accounts of people stumbling and falling after drinking. Alcohol, even at these low concentrations, affects brain function and increases fall risk. An increased fall risk has been associated with impaired obstacle avoidance skills. Low level BACs are likely to affect obstacle avoidance reactions during gait, since the brain areas that are presumably involved in these reactions have been shown to be influenced by alcohol. Therefore we investigated the effect of low to moderate alcohol consumption on such reactions.

Thirteen healthy senior individuals (mean(SD) age: 61.5(4.4) years, 9 male) were subjected to an obstacle avoidance task on a treadmill after low alcohol consumption. Fast stepping adjustments were required to successfully avoid suddenly appearing obstacles. Response times and amplitudes of the m. biceps femoris, a prime mover, as well as avoidance failure rates were assessed.

**Findings:**

After the first alcoholic drink, 12 of the 13 participants already had slower responses. Without exception, all participants' biceps femoris response times were delayed after the final alcoholic drink (avg ± sd:180 ± 20 ms; *p *< 0.001) compared to when participants were sober (156 ± 16 ms). Biceps femoris response times were significantly delayed from BACs of 0.035% onwards and were strongly associated with increasing levels of BAC (*r *= 0.6; *p *< 0.001). These delays had important behavioural consequences. Chances of hitting the obstacle were doubled with increased BACs.

**Conclusions:**

The present results clearly show that even with BACs considered to be safe for driving, obstacle avoidance reactions are inadequate, late, and too small. This is likely to contribute to an increased fall risk. Therefore we suggest that many of the alcohol-related falls are the result of the disruptive effects of alcohol on the online corrections of the ongoing gait pattern when walking under challenging conditions.

## Background

Alcohol is a commonly used social drug and driving under influence is a well-established risk factor for traffic accidents [1]. To improve road safety, legal limits are set for blood alcohol concentration (BAC) and driving, usually at 0.05% (most European countries) or 0.08% (most US states, Canada and UK). For other tasks than driving, however, it remains unclear whether these BACs also reflect appropriate safety limits. Recent research showed that among working-aged people, ingestion of alcohol in the previous 6 hours is strongly and consistently related to falls at home resulting in admission to hospital or death, even with low levels of alcohol consumption [2]. The public health impact of falls is substantial and concomitant costs are growing [2,3]. However, reducing alcohol intake is often not included in intervention strategies to prevent falls. Low alcohol intake is generally not deemed unsafe with regard to falls, but this was never investigated systematically. Accidental falls have been found to be associated with impaired obstacle avoidance skills [4]. To prevent tripping, accurate goal-directed reactions are required to avoid sudden obstacles in the travel path. In previous work we have observed that an increased percentage of obstacles that were hit ("obstacle avoidance failures") is associated with the presence of smaller and later EMG responses in the prime movers (such as the m. biceps femoris) involved in the obstacle avoidance reaction [5,6]. From cat studies it is known that the parietal cortex and the cerebellum play an important role in this reaction [7,8]. Imaging studies have shown that acute alcohol administration significantly reduces brain glucose metabolism in these areas that are important for obstacle avoidance [9-11]. Hence, one would predict obstacle avoidance reactions during gait to be disturbed by alcohol ingestion. Therefore we investigated the effect of low to moderate alcohol consumption on such reactions in healthy senior individuals by means of an obstacle avoidance task.

It was hypothesized that obstacle avoidance reactions are already impaired at low BACs, and that the increases in the percentage of obstacles that were hit after alcohol consumption will be accompanied by delayed and decreased muscle responses in the m. biceps femoris.

## Methods

### Participants

Thirteen healthy senior individuals (mean(SD) age: 61.5(4.4) years, 9 male) volunteered to participate in this study. None of the participants was, or used to be a habitual drinker. Inclusion criteria were absence of any known serious neurological, orthopaedic or cognitive dysfunction, and age between 50-70 years. Exclusion criteria were a bodyweight exceeding 100 kg or the use of (prescribed) medication(s) that could interfere with alcohol. As the experiment took place in the late afternoon, participants were instructed to just have an early light lunch (e.g. a sandwich), and not to drink caffeinated drinks in the 4 hours before arriving at the laboratory. Subjects were informed about the experimental procedure before they gave their written informed consent in accordance with the ethical standards of the Declaration of Helsinki. The protocol was approved by the ethical committee of the region Arnhem-Nijmegen.

### Equipment and procedure

**Figure 1:**
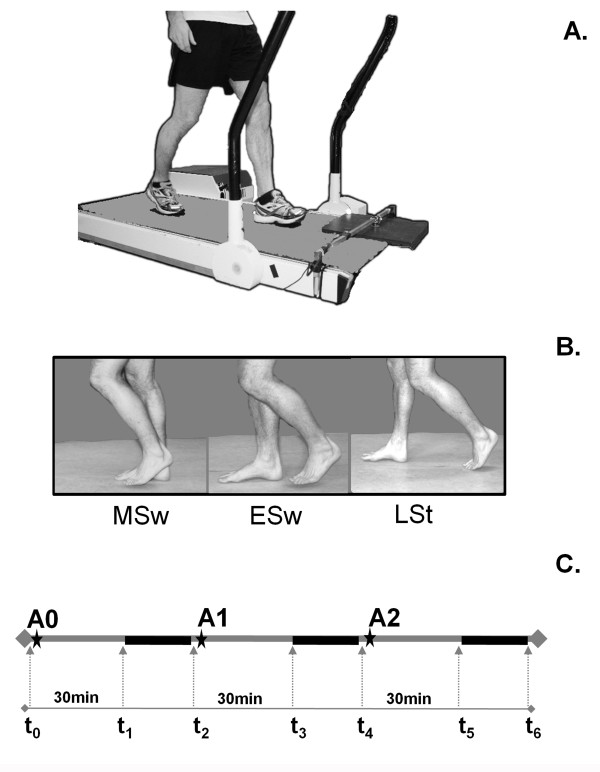
**Methods**. **A**. Experimental setup. **B**. Step cycle phases in which the obstacle was released. LSt = Late Stance, ESw = Early Swing, MSw = Mid Swing **C**. Protocol: assessment of BAC at t0-t6. A0: placebo, A1: first alcoholic drink, A2: second alcoholic drink. Solid black line: obstacle avoidance task.

The participants were instructed to avoid obstacles while walking on a treadmill (ENRAF Nonius, Type EN-tred Reha) at a fixed velocity of 3 km/hr (Figure 1A), wearing their own comfortable shoes (no high heels). A wooden obstacle (measuring 40x30x1.5cm) with an embedded piece of iron was held by an electromagnet just above the treadmill surface. Its release could be triggered by the computer. The obstacle was always presented to the left foot. On both feet, three reflective markers (diameter 14 mm) were attached at heel, hallux and lateral malleolus. A single marker was placed on top of the obstacle. Marker positions were recorded by an 8-camera 3-D motion analysis system (Vicon^®^, Oxford Metrics, London, UK) at a sample rate of 100 Hz. The marker positions were processed in real time in order to determine the moment of obstacle release related to gait phase. The real time processing also enabled the experimenter to check online the foot position with respect to the obstacle, while the participants were instructed to walk at a fixed distance to the obstacle that was approximately 10 cm from the most anterior position reached by the toes in the swing phase. If they deviated more than 3 cm from this position, participants received verbal feedback to correct the distance to the obstacle. The obstacle was not released until a regular walking pattern was observed and until at least five unperturbed strides for the trial had been completed. Stride regularity was defined as a maximum difference of 50 ms between two consecutive strides. The obstacle was dropped at one of three different phases of the step cycle (late stance (*LSt*,45-60% of the step cycle), early swing (*ESw*, 60-70%) or mid swing (*MSw*,70-85%)) to create different levels of difficulty to avoid the obstacle as time pressure increased (Figure 1B). Available response time (ART), the measure of time pressure, was defined as the time between obstacle release and the estimated moment of foot contact with the obstacle if no adjustment of the stride had been made [12]. The obstacle release phases corresponded with ARTs greater than 450 ms (*LSt*), 300-450 ms (*ESw*), and 150-300 ms (*MSw*). Ten obstacles in each of the three phases of the gait cycle were presented in random order during a series of 30 trials.

The participants were instructed to look at the obstacle, and step over it after its release. Stepping to the side was discouraged, and any contact of the left foot with the obstacle was defined as a failure. Since the m. biceps femoris (BF) is known to be the prime mover involved in the avoidance reaction [6], surface electromyography (EMG) data were collected from this muscle to assess response times. Self-adhesive Ag-AgCl electrodes (Tyco Arbo ECG) were placed approximately 2 cm apart and longitudinally on the belly of the muscle, according to European guidelines [13]. The EMG signals were sampled at 2400 Hz (ZeroWire^®^, Aurion S.r.l., Italy) and recorded synchronously with the marker data.

Three series of 30 obstacle avoidance trials were performed, each 30 minutes after ingestion of a drink (Figure 1C). Subjects were informed that these drinks contained alcohol, and had to finish them within 10 minutes. The first (*A0*) was a placebo consisting of water mixed with orange juice (ratio 1:3) with a drop of vodka floated on top to give the scent of alcohol. The following two drinks (*A1 *and *A2*) each contained 40% vodka mixed with orange juice (ratio 1:3). We aimed to reach a BAC that was around the common legal limits for driving (0.05% for most European countries or 0.08% for most US states, Canada and UK) 30 minutes after A2, having used the Widmark formula [14] to adjust the alcohol dosage for the individual's gender and weight. A Dräger Alcotest^® ^7410 Plus com breathalyzer was used to determine the BAC before, during, and after the experimental task (Figure 1C). For safety reasons, all participants were taken home by a taxi after the experiment was finished.

### Data analysis

Successful obstacle avoidance for each trial was scored. This was easily determined by two observers by eye, and by feedback from the participant. If there was any doubt about the successfulness, the marker data were checked (this happened in less than 1% of the cases). As the primary outcome measure, failure rates (as defined by the number of failed trials divided by the total number of trials) were calculated for each alcohol condition and each step cycle phase.

**Figure 2:**
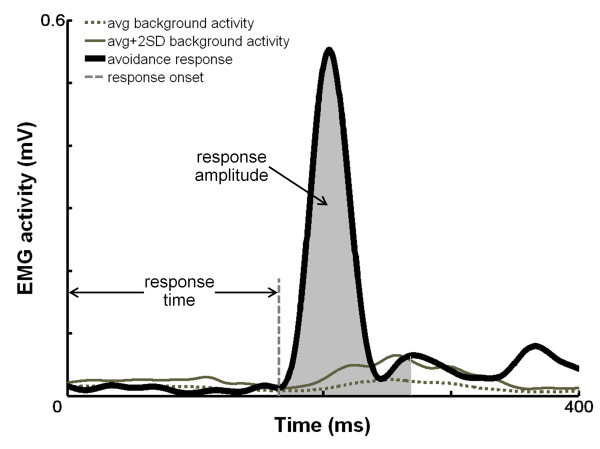
**Determination of response time and amplitude of the m. biceps femoris (BF)**. Response time was defined as the time between obstacle release (set at the origin of the axes) and the moment where the BF activity exceeded the activity of the control stride + 2SD. Average response amplitude was calculated over 100 ms after the onset of the response and normalized with respect to the average background activity.

To assess the EMG responses, the EMG activity of the m. biceps femoris (BF) was full-wave rectified and low-pass filtered at 25 Hz (zero lag, 4^th ^order Butterworth filter). Background EMG was calculated for each series separately as the average BF activity over 25 control strides (i.e. the stride preceding that in which obstacle release occurred). For each participant and alcohol condition, BF response times were determined as the time between obstacle release and the moment at which BF activity exceeded the average control stride by at least 2 SDs for more than 30 ms (for example, see Figure 2). This was done with the help of a custom made computer algorithm (Matlab^® ^software, version 7.4.0, The Mathworks Inc., US). Each trial for which a response time was calculated was visually checked for correct determination of the response onset. In about 2% of the trials the onsets were corrected. The onsets of the avoidance responses for each subject were averaged for each phase of obstacle release within each alcohol condition. The responses amplitude was calculated as the average amplitude during the 100 ms following the onset of the BF response [5,15]. The amplitudes were normalized with respect to the maximum average background activity during the whole step cycle in the A0 condition. A similar procedure was performed to calculate and normalize the average control stride activity in the 100 ms following the BF response onset.

### Statistical analysis

To check whether within participants, the series were equally difficult in the three alcohol conditions, we compared the average ARTs in a repeated measures MANOVA (within-subjects factors: alcohol condition (A0, A1, A2); phase of obstacle release (LSt, ESw, MSw), α = 0.05) with post-hoc pairwise comparisons.

To identify the effect of BAC on avoidance failure rates we used a binary logistic regression with alcohol condition as categorical factor (A0 as reference category, α = 0.05) in Egret^® ^for Windows (version 2.0.31). A statistical model was fitted to the data of the MSw phase to predict the probability of a failure with increasing BAC for the most time critical situations.

For the analysis of EMG data, we used repeated measures MANOVAs with post-hoc pairwise comparisons to test for differences between the three alcohol conditions (within subjects-factor: A0, A1, A2; α = 0.05) for average BF response times, normalized response amplitudes, and normalized background activity. The relationship between BF response time and BAC was assessed by means of bivariate correlation (Pearson Correlation Coefficient). One sample Students' t-test with bins of 0.005% BAC was used to determine the BAC from which the BF response time was significantly delayed. These analyses were carried out in SPSS^® ^(version 12.0.1) with α set at 0.05. Means are presented with their standard errors (SE).

Pilot data indicated that the difference in obstacle avoidance response time after 2-3 standard alcoholic drinks was 20 ms (SD: 18 ms). To be able to identify a difference of 20 ms in the mean response time between A0 and A2, a sample size of 11 subjects would be needed in this repeated measures design (β = 0.9, α = 0.05).

## Results

Before the start of the experiment, the BAC of each participant was 0.00%. After the final drink the BACs found ranged from 0.03% to 0.06%. Hence, we succeeded in our aim to reach BACs around or below common legal limits for driving (0.05% for most European countries or 0.08% for most US states, Canada and the UK).

The series of the obstacle avoidance trials were equally difficult in the three alcohol conditions (A0, A1, and A2), as there were no significant main effects of alcohol condition on the average ARTs (*F_2,11 _*= 0.22, *p *= 0.81).

### Failures

**Figure 3:**
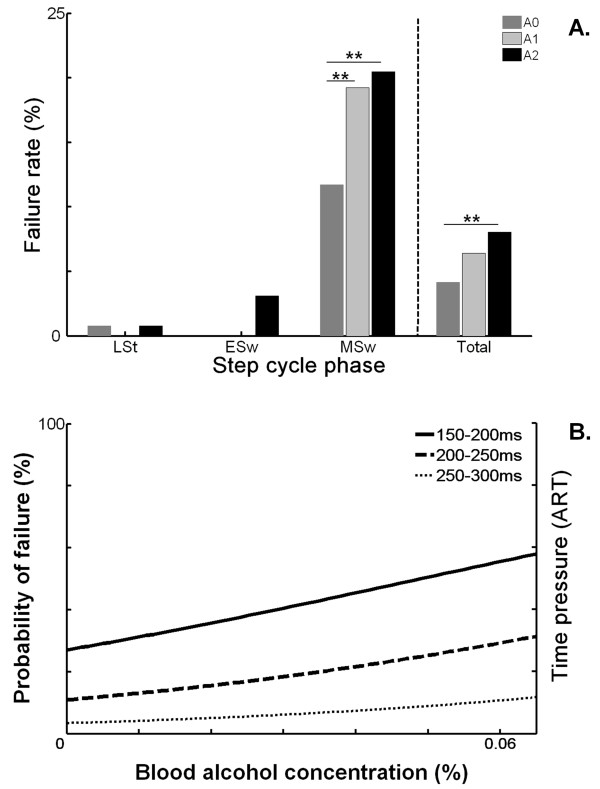
**Effect of alcohol on failures**. **A **. Failure rate per alcoholic condition for each step cycle phase separately, and in total. LSt = Late Stance, ESw = Early Swing, MSw = Mid Swing. A0: placebo, A1: first alcohol, A2: second alcohol. (**p < 0.01, compared to A0). **B**. Model of the probability of failing to avoid the obstacle with increasing blood alcohol concentrations for the most time critical situations.

Figure 3A shows the effect of alcohol on avoidance failure rates. Overall, the failure rate increased significantly from 4.5% (A0) to 8.8% (*p *< 0.01) after consumption of two alcoholic drinks. Figure 3A also demonstrates that in each alcohol condition most failures were made in the MSw phase (*p *< 0.01, compared to late stance). Within this phase, the failure rate increased significantly with alcohol consumption, from 11.7% in A0, to 19.2% and 20.5% in A1 and A2, respectively (*p *< 0.01, compared to A0). Moreover, the probability of a failure not only increased with higher BACs, but also with lower ARTs, which corresponded to the MSw phase (Figure 3B). Compared to the placebo condition, chances of hitting the obstacle almost doubled after the final alcoholic drink (*odds ratio (95%CI) *= 1.93 (1.17,3.18); *p *= 0.01).

### Response time

**Figure 4:**
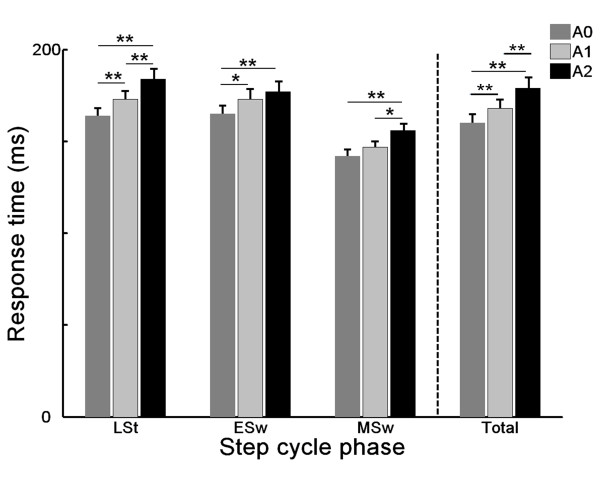
**Effect of alcohol on BF response times for each step cycle phase separately, and in total**. LSt = Late Stance, Esw = Early Swing, MSw = Mid Swing. A0: placebo, A1: first alcohol, A2: second alcohol. (*p < 0.05, **p < 0.01)

**Figure 5:**
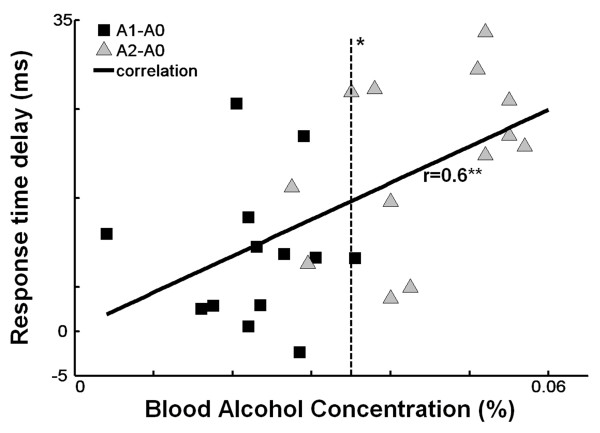
**Individual delays in BF response times after the first (A1) and second (A2) drink as compared with the A0 condition.** Each data point represents one subject in the corresponding alcohol condition. The solid line shows the correlation between BAC and the delay in response time (**p < 0.01). The dashed line represents the BAC from whereon the delay is significant (*p < 0.05).

The results for BF response times, one of the proposed determinants of successful obstacle avoidance, are shown in Figure 4. Repeated measures MANOVA revealed a main effect of alcohol condition on overall BF response times (*F_2,11 _*= 24.93, *p *< 0.001), as well as for each phase of obstacle release separately (Table 1). After ingestion of 2 alcoholic drinks (mean ± SD: 0.47 ± 0.04 g alcohol/kg bodyweight), BF response times were delayed by 12% compared to when participants were sober (179 ± 5.8 vs 160 ± 4.7 ms, *F_1,12 _*= 53.42, *p *< 0.001) (Figure 4). From the data of the individual subjects (Figure 5) it can be seen that without exception, BF response times for all participants were delayed following A2. Furthermore, even after A1, 12 of the 13 participants already responded more slowly. The BF response times were significantly delayed from BACs of 0.035% upwards (*t *= 18.05; *p *= 0.003). There was a significant correlation between response time and level of BAC (*r *= 0.6; *p *< 0.001; Figure 5).

**Table 1:** Repeated measures MANOVA with within subjects contrast for BF response latencies

	LSt			ESw			MSw			Total		
	*Diff(ms)*	*F_1,12_*	*p*	*Diff(ms)*	*F_1,12_*	*p*	*Diff(ms)*	*F_1,12_*	*p*	*Diff(ms)*	*F_1,12_*	*p*
A0 vs A1	8.3	11.0	**0.006**	9.2	5.6	**0.036**	5.4	2.0	0.182	8.7	15.0	**0.002**
A1 vs A2	10.8	12.0	**0.005**	5.0	1.2	0.287	11.0	18.4	**0.001**	10.8	22.7	**0.000**
A2 vs A0	19.2	26.8	**0.000**	14.2	10.0	**0.008**	16.4	36.3	**0.000**	19.4	53.4	**0.000**

A0 = placebo, A1 = first alcohol, A2 = second alcohol, LSt = Late Stance, ESw = Early Swing, MSw = Mid Swing, Total = all trials, Diff = difference. Bold numbers indicate significance.

A significant effect of obstacle release phase on BF response times was also found. The LSt responses were significantly slower than those in ESw (*F_1,12 _*= 52.65, *p *< 0.001). In turn, ESw responses were again significantly slower than those in MSw (*F_1,12 _*= 49.86, *p *< 0.001).

### Response amplitude

**Figure 6:**
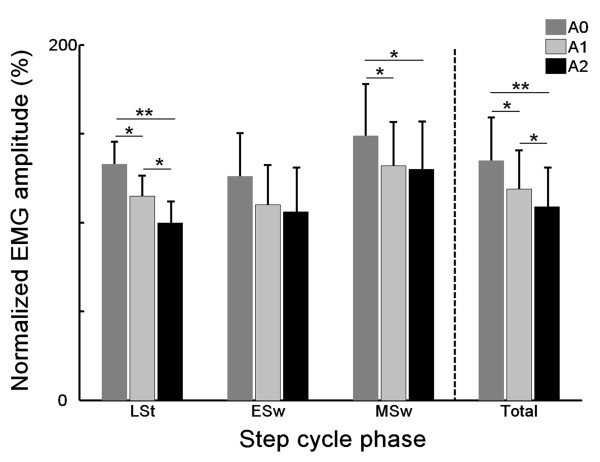
**Effect of alcohol on normalized EMG amplitudes for the m. biceps femoris**. LSt = Late Stance, Esw = Early Swing, MSw = Mid Swing. A0: placebo, A1: first alcohol, A2: second alcohol. (*p < 0.05, **p<0.01).

Figure 6 shows the results of the normalized response amplitudes. Repeated measures MANOVA revealed a main effect of alcohol condition on overall BF response amplitudes (*F_2,11 _*= 4.83, *p *= 0.03). Post-hoc analyses yielded a significant effect of alcohol condition on response time in LSt, MSw, and in total (Table 2). A trend for decreasing amplitude with increasing BACs can be noted in all step cycle phases (Figure 6).

**Table 2:** Repeated measures MANOVA with within subjects contrast for BF response amplitudes

	LSt			ESw			MSw			Total		
	*Diff(%)*	*F_1,12_*	*P*	*Diff(%)*	*F_1,12_*	*p*	*Diff(%)*	*F_1,12_*	*p*	*Diff(%)*	*F_1,12_*	*p*
A0 vs A1	18.4	3.5	0.087	15.8	4.6	0.054	17.1	4.7	**0.050**	16.5	9.1	**0.011**
A1 vs A2	14.7	11.2	**0.006**	4.2	2.3	0.158	1.5	2.5	0.142	10.1	9.1	**0.011**
A2 vs A0	33.1	8.3	**0.014**	20	4.7	0.052	18.6	5.7	**0.035**	26.5	10.3	**0.007**

A0 = placebo, A1 = first alcohol, A2 = second alcohol, LSt = Late Stance, ESw = Early Swing, MSw = Mid Swing, Total = all trials, Diff = difference. Bold numbers indicate significance.

To rule out the possible effect of background activity on amplitude, the background activity was analyzed as well. The normalized background activity did not change significantly with increasing BACs (*F_2,11_*= 0.24, *p *= 0.79).

## Discussion

The present study investigated the effect of low to moderate levels of alcohol consumption on obstacle avoidance reactions during gait. The results clearly show that even with low BACs ( < 0.06%), reactions to sudden gait perturbations are seriously affected. After ingestion of 2 alcoholic drinks, obstacles were hit more often, BF response times were delayed and response amplitudes were reduced. These changes were most obvious in situations with little available response time.

This is the first study to investigate the effect of alcohol on responses to sudden gait perturbations (as a relevant task related of falls). Previous studies have concentrated on the effects on posture [16,17]. Low doses usually increased body sway but in some subjects the inverse was seen, indicating that these doses may have an beneficial effect in some cases [17]. However, it is questionable whether these data are actually relevant for falls since falls rarely occur during standing. Locomotion studies seem to be more relevant in this respect. Mallinson et al.[18] found that it may be possible to detect subtle dynamic imbalance induced by alcohol ingestion (89 ml of 40% alcohol) during tandem walking with eyes closed. In the present study participants had to walk on a treadmill with eyes open after consuming an average of 125 ml of 40% alcohol. Because the background activity was not significantly different between the alcohol conditions, and the obstacle was only released when a regular walking pattern was observed and after at least five unperturbed strides had been taken from the start of the trial, we feel confident that any differences found in the failure rate and any changes in BF activity reflect the effect of the increased BAC.

Earlier research has shown that many falls are primarily due to stumbling and tripping [19]. In order to avoid falls due to hitting an obstacle, one needs to be able to respond adequately to both unseen obstacles causing a stumble [20,21], and to obstacles suddenly appearing in the travel path [6]. The muscle that shows the first major response in all these reactions is the m. biceps femoris [6,15,20,21]. When compared to young adults, both an increase in response latency and a decrease in response amplitudes of this muscle were found in older adults [6,22]. These longer onset latencies and smaller amplitudes were associated with lower success rates [6]. The underlying mechanism for the decreased amplitudes during the stumbling and obstacle avoidance reactions in older adults may involve various age-related physiological changes, both in the CNS (e.g. fewer motoneurons) [23] and in skeletal muscle properties (fewer type II muscle fibers and overall muscle atrophy) [23,24]. In contrast, in the present study the delay and decrease in response are more likely to be due to CNS changes. A possible explanation for the increased failure rate could be that the pathways used in the avoidance reactions have been altered by alcohol consumption. Obstacle avoidance reactions are often very fast; this has led to the proposal that fast supraspinal pathways may be involved that bypass the primary motor cortex [6,25]. These pathways may involve the parietal cortex and/or the cerebellum. For example in studies on cats, Drew [26] showed that the parietal cortex is involved in obstacle avoidance. They also proposed that both a fast directly descending pathway originating from the parietal cortex may exist along with a slower one involving the motor cortex.

Another possible explanation for the impaired obstacle avoidance skills after alcohol consumption lies at the level of neurotransmitters. For example, previous research has shown that the endorphinergic system [27] and GABA (Gamma-AminoButyric Acid) [28] are intimately involved in the actions of alcohol. The sedative, tranquilizing and/or anaesthetic properties of alcohol have been related to the enhancement of the flow of chlorine ions across neural membranes due to GABA [28]. Yet alcohol does not have this effect on all GABA receptors. Motor incoordination due to ethanol is caused by potentiation of GABA_A_-associated adenosine A_2A _receptors in the striatum (caudate nucleus & putamen) [28]. Moreover, it is suggested that alcohol-induced deterioration in motor function is linked to changes in patterns of brain activity rather than changes in specific brain regions. Specifically, changed activity in the cerebellum as well as in the frontal and parietal cortices are involved in the motor-incoordinating effects of alcohol [29].

Studies on the effects of alcohol on brain activity with leg movements are lacking. However, for arm movements Van Horn et al. [30] found that the human cerebellum and PPC (Posterior Parietal Cortex) are involved in goal-oriented limb movements and that this role is compromised by alcohol. In particular, alcohol may cause a disturbance in the ability of these brain regions to compute appropriate corrective behavioral responses [30]. In this context it is reasonable to suggest that the presently observed deficits in obstacle avoidance skills may be due to the effect of alcohol on information processing in the PPC and the cerebellum. Experiments involving techniques to record brain activity during obstacle avoidance should be performed to test this hypothesis.

## Limitations

To limit discomfort to the subjects we used a handheld breath analyzer instead of blood samples. In contrast to blood analysis, these breath analyzers are easy and quick to use, do not require additional hard or software, and are not a burden to the participants. The readings of such portable devices are in good agreement with the results of confirmatory analyses performed by stationary devices (*r *= 0.978) [31]. Furthermore, the correlation with blood analysis is quite high for both the readings of the handheld (*r *= 0.940) as well as the stationary devices(*r *= 0.936) [31].

To the best of our knowledge, the effect of alcohol or other substances on obstacle avoidance during gait has never been studied before in healthy senior individuals. Therefore, it is not possible to make a direct comparison with results from similar studies. However, the obstacle avoidance task used in the present study has proven to be sensitive enough to detect significant age-related deficits [32]. A possible limitation is the relatively small sample size. However, in this type of motor control studies it is quite usual to have similar group sizes (because of the extensive data analysis involved). Furthermore, even with the small number the study yielded unequivocal outcomes. Hence, a larger sample size will mostly accentuate the significance of the present results.

## Conclusions

In conclusion, the present results clearly show that alcohol levels, considered to be safe for driving, seriously hamper the ability to successfully avoid sudden obstacles in the travel path. It is suggested that many of the alcohol-related falls are the result of the disruptive effects of alcohol on the online corrections of the ongoing gait pattern when walking under challenging conditions. In general the use of alcohol is primarily seen as a risk factor for driving [1,33]. However, Kool et al. [2] estimated that approximately 20% of unintentional falls at home in a working-aged population may be attributable to the consumption of two or more standard alcoholic drinks in the preceding 6 h. Moreover, accidents can also occur while walking, particularly under challenging conditions such as when negotiating suddenly appearing obstacles. The present data show that the required skills for obstacle avoidance frequently fail even after consumption of a low dose of alcohol.

## List of abbreviations

**BAC**: Blood Alcohol Concentration; **LSt**: Late Stance; **ESw**: Early Swing; **MSw**: Mid Swing; **ART**: Available Response Time (the time between obstacle release and the estimated moment of foot contact with the obstacle if no adjustment of the stride had been made (Chen et al., 1994)); **BF**: m. Biceps Femoris; **A0**: placebo; **A1**: first alcohol; **A2**: second alcohol.

## Competing interests

The authors declare that they have no competing interests.

## Authors' contributions

JH, VW, BB and BN conceived and designed the study. JH recruited the participants, performed the experiment and the analysis, and made the first draft of the manuscript. BN gave technical support. JL played a major role in the statistical analysis. VW, BB, BN, JL and JD contributed to the analysis and interpretation of the data, and revised the manuscript critically. All authors read and approved the final manuscript.
